# Multinucleation followed by an acytokinetic cell division in myxofibrosarcoma with giant cell proliferation

**DOI:** 10.1186/1756-9966-28-44

**Published:** 2009-03-31

**Authors:** Takashi Ariizumi, Akira Ogose, Hiroyuki Kawashima, Tetsuo Hotta, Hajime Umezu, Naoto Endo

**Affiliations:** 1 Division of Orthopedic Surgery, Niigata University Graduate School of Medical and Dental Sciences, Niigata, Japan; 2 Division of Pathology, Niigata University Hospital, Niigata, Japan

## Abstract

**Background:**

Multinucleated cells are frequently seen in association with a malignant neoplasm. Some of these multinucleated cells are considered to be neoplastic. The mechanism of neoplastic multinucleation remains unknown, but is considered to be induced by either cell-cell fusion or acytokinetic cell division. Myxofibrosarcoma consists of spindled and pleomorphic tumor cells and bizarre multinucleated giant cells. Some of these multinucleated cells are considered to be neoplastic.

**Methods:**

We studied the mitotic activity of the multinucleated cells by Ki-67 immunohistochemistry, and the dynamics and differentiation by live-cell video microscopy in the two myxofibrosarcoma cell lines to determine whether the mechanism of multinucleation is cell-cell fusion or acytokinetic cell division

**Results:**

A Ki-67 immunohistochemical analysis revealed a high positive rate of multinucleated cells, as well as mononuclear cells, and mitotic ability was shown in the multinucleated cells. In live-cell video microscopy, most of the multinucleated cells were induced via the process of acytokinetic cell division.

**Conclusion:**

The current study indicates that a vulnerability of the cytoskeleton components, such as the contractile ring, causes multinucleation to occur from the telophase to the cytokinesis of the cell cycle.

## Background

A multinucleated cell is a unique form which is frequently observed in the normal tissue. Skeletal muscle is composed of bundles of multinucleate muscle fibers [1]. Osteoclasts induce multinucleation by the cell fusion of mononuclear cells to cover a large area for bone resorption [2]. Macrophages may fuse to form multinuclear giant cells when adequately stimulated [3]. Many hepatocytes are binucleate, and the nuclei are frequently polyploidy [4].

On the other hand, multinucleated cells are frequently seen in malignant neoplasms. Giant cells may be formed and possess either one enormous nucleus or several nuclei [5]. In Hodgkin's disease, Reed-Sternberg cells have an intricate double or bi-lobed nucleus [6].

The mechanism of neoplastic multinucleation remains unknown, but is considered to be induced by cell-cell fusion or acytokinetic cell division.

Myxofibrosarcoma is one of the most common sarcomas in elderly patients with a slight male predominance and this tumor consists of spindled and pleomorphic tumor cells and bizarre multinucleated giant cells with abundant eosinophilic cytoplasm [7]. Some of these multinucleated cells are considered to be neoplastic and possess atypical nuclei or mitotic changes [8]. However, it is not known precisely by what mechanism multinucleated cells are formed. To determine whether the mechanism of multinucleation is cell-cell fusion or acytokinetic cell division, we elucidated the activity of the multinucleated cells by Ki-67 immunohistochemistry and the dynamics and differentiation by live-cell video microscopy in the two myxofibrosarcoma cell lines.

## Methods

### Tumor cell lines

The human myxofibrosarcoma cell lines, NMFH-1 and NMFH-2, were used for these experiments. NMFH-1 was described previously [9]. NMFH-2 has been newly established in our institute. The cell line originates from the soft tissue tumor of the left upper arm of a 79-year-old male. Histologically, the tumor was comprised of spindle shaped cells and multinucleated giant cells partially forming storiform pattern. These cell lines were maintained in a culture medium (RPMI 1640) supplemented with 10% FBS, 0.6% Kanamycin Sulfate (GIBCO, Grand Island, NY), and 1% Antibiotic-Antimycotic (GIBCO, Grand Island, NY). The parental tumours of these two cell lines were fixed with formalin and embedded with paraffin. The paraffin embedded-specimens were cut into 4 μm thick sections and then were evaluated immunohistochemically.

### Tumor implantation in SCID mice

NMFH-1 cells (5 × 10^6^) derived from 100-time passages and NMFH-2 cells (5 × 10^6^) derived from 30-time passages were injected subcutaneously into the backs of 7-week-old female athymic SCID mice (CB-17/Icr scid; Jcl CLEA Japan, Inc., Osaka, Japan). The transplanted tumors were successfully formed and these xenografted tumors were fixed with formalin and embedded with paraffin. Paraffin embedded-specimens were then cut into 4 μm thick sections and analyzed immunohistochemically.

### Ki-67 immunohistochemistry

Ki-67, bromodeoxy-uridine (BrdU) and proliferating cell nuclear antigen (PCNA) were useful for proliferative markers. BrdU was diffucult to inject into the parental tumors. PCNA showed non-specific reactions in the cytoplasms of the cultured cells in our pilot study. We therefore examined Ki-67 immunohistochemistry for the proliferation of both mononuclear and multinucleated cells. Briefly, both types of cultured cells were incubated on Lab-Tek chamber slides (Nalge Nunc International, Rochester, NY, USA), fixed with 100% methanol for 10 min. The sections of parental tumors and xenografts were deparaffinized in xylene, and then were rehydrated gradually, and heated at 100°C for 20 min with 10 mM citrate buffer (pH6.0) for antigen retrieval. Next, the specimens were treated with 0.3% hydrogen peroxide in methanol for 20 min to inhibit endogenous peroxidase, and incubated with phosphate-buffered saline containing 10% goat serum (Dako, Denmark) for 30 min to reduce nonspecific reactions. The specimens were then incubated with the monoclonal mouse antibody (MIB-1, Dako, Denmark) diluted 1:100 for 60 min, and reacted for 60 min with peroxidase-labeled anti-rabbit or anti-mouse antibody (Histofine Simple Stain MAX PO (MULTI); Nichirei Corporation, Tokyo, Japan) for 60 min. All these procedures were performed at room temperature. The peroxidase activity was detected with 3'-diaminobenzidine tetrahydrochloride (Nichirei, Tokyo, Japan). The specimens were counterstained with hematoxylin.

### The live cell observation

Time-lapse video microscopy was used in this experiment. This system has an incubator with a built-in microscope to observe and record the real-time motion of the live cells in the incubator. Both cell types were separately incubated on the non-coated culture dishes, and placed in the incubation imaging system (LCV100, Olympus, Tokyo, Japan) [10,11]. The cultured cells were maintained in 5% CO_2 _plus 95% air at 37°C in the incubator, and then were observed and photographed at intervals of 15 min for 72 hours. The recorded pictures were converted to the animation. All cells which came in sight at the start of observation were traced, and the morphological alteration of each cell was examined.

## Results

### Histologic findings of the cultured cells, parental tumors and xenografts

**Figure 1:**
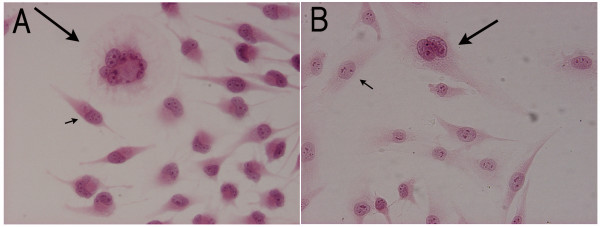
**Hematoxylin and eosin staining of the cultured NMFH-1 and NMFH-2 cells**. These were composed of spindle shaped mononuclear cells (short arrow), and multinucleated giant cells (long arrow). A: NMFH-1 B: NMFH-2 (magnification, × 200).

**Figure 2:**
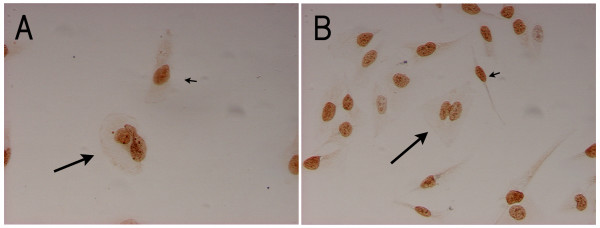
**Ki-67 immunohistochemistry of the cultured NMFH-1 and NMFH-2 cells**. Most of the multinucleated cells were positive for Ki-67 (long arrow), as were most of the spindle mononuclear cells (short arrow). A: NMFH-1 B: NMFH-2 (magnification, × 200)

**Figure 3:**
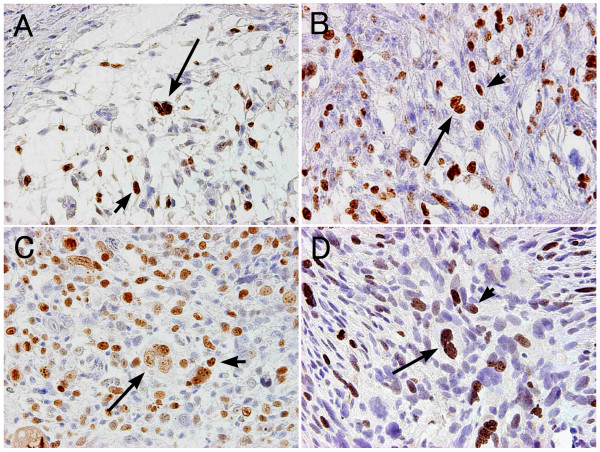
**Ki-67 immunohistochemistry of the parental tumors and xenografts of the NMFH-1 and NMFH-2 cells**. Regarding the parental tumors and xenografts, focal reactivity was present in multinucleated cells (long arrow) as well as the mononuclear cells (short arrow). A: The parental tumor of NMFH-1 cells B: The parental tumor of NMFH-2 cells. C: Xenograft of NMFH-1 cells D:Xenograft of NMFH-2 cells (magnification, × 200).

Both cultured cells showed the same pattern of cell forms. These were composed of two different cell types: spindle shaped mononuclear cells and multinucleated giant cells (Figure 1). The frequency of multinucleated cells was fewer than that of mononuclear cells, and only 5.00% of the NMFH-1 cells and 10.2% of the NMFH-2 cells were multinucleated. As shown by Ki-67 immunohistochemistry, not only were most of the spindle-shaped mononuclear cells positive for Ki-67, but most of the multinucleated cells were also positive (Figure 2). Both cells showed a high Ki-67 positive rate. In NMFH-1, the Ki-67 positive rate was 93.9% of the mononuclear cells and 84.9% of the multinucleated cells. In the NMFH-2 cells, the Ki-67 positive rate was 90.4% of the mononuclear cells and 80.8% of the multinucleated cells (Table 1). Regarding the parental tumors, focal reactivity was present in multinucleated cells as well as mononuclear cells (Figure 3-A, B). In the tumor xenografts, a portion of the multinucleated cells also expressed Ki-67 (Figure 3-C, D).

**Table 1:** Ki-67 positive rate

Ki-67 positive rate (%)	NMFH-1	NMFH-2
Mononuclear cell	93.9	90.4
Multinucleated cell	84.9	80.8

### Dynamics and differentiation of the live cells

A total of 226 NMFH-1 cells and 50 NMFH-2 cells were found at the start of the live-cell observation. There were 2 multinucleated cells in the NMFH-1 observation and 4 multinucleated cells in the NMFH-2 observation. All of these cells were traced and verified for 72 hours.

At the last observation, the total cell count was 687 for the NMFH-1 cells and 106 for the NMFH-2 cells. The average doubling times were 44 hours in the NMFH-1cells, and 455 hours in the NMFH-2 cells. During this period, normal cell division was observed 342 times in the NMFH-1cells, and 70 times in the NMFH-2 cells, and multinucleation was observed 83 times in the NMFH-1cells, and 16 times in the NMFH-2 cells.

**Figure 4:**
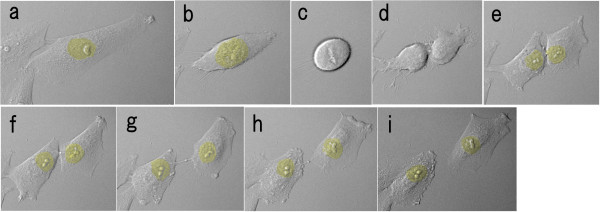
**Dynamics of normal cell division by time-lapse video microscopy**. The interval of each image is 15 minutes. These images were taken by the incubation imaging system, LCV100, Olympus. The yellow area indicates the location of the nuclei. From anaphase to cytokinesis, the constriction proceeded and the cytoplasm of the parent cell was divided, and then the daughter cytoplasms did not fuse.

Regarding normal cell division, which arose in a large number of the traced cells, the constriction proceeded and the cytoplasm of the parent cell was divided and then the daughter cytoplasm did not fuse from the anaphase to cytokinesis (Figure 4; Additional file 1).

**Figure 5:**
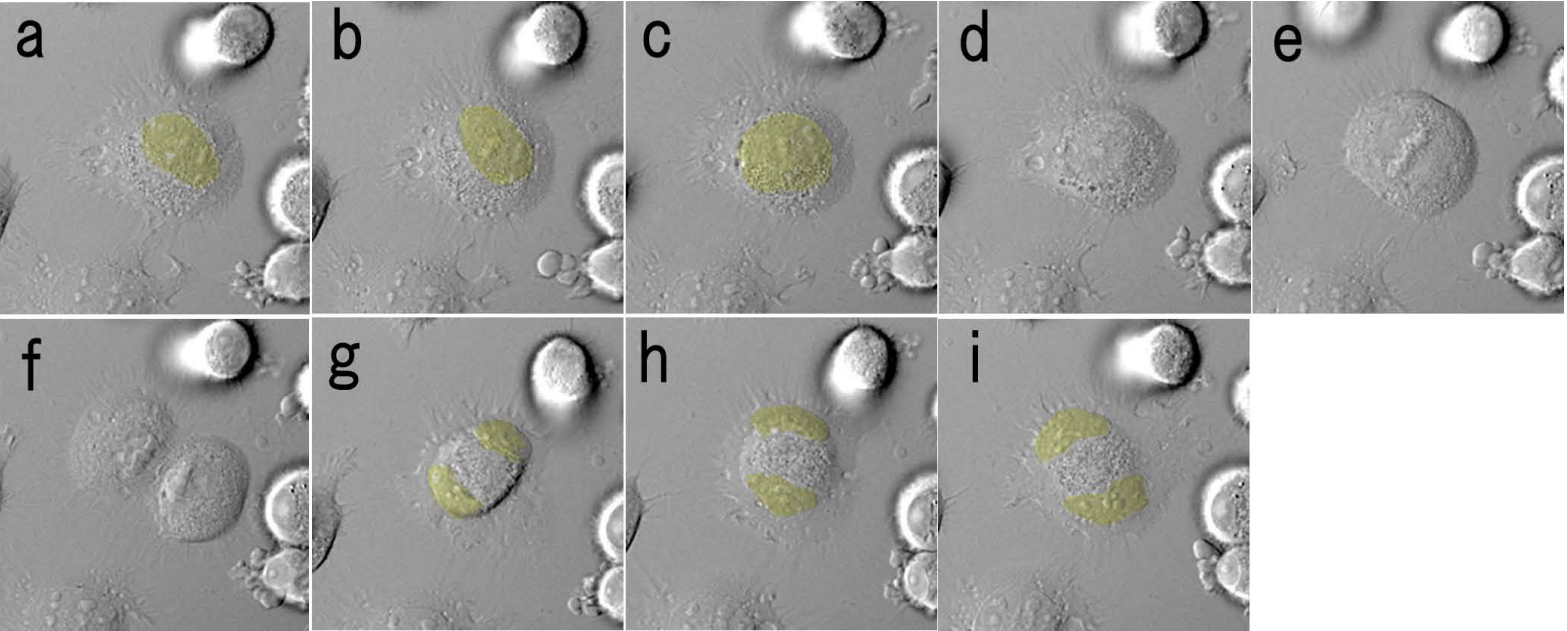
**Dynamics of multinucleation by time-lapse video microscopy**. The interval of each image is 15 minutes. The yellow area indicates the location of the nuclei. In prophase, the nuclear body and the nuclear membrane were disaggregated and could not be seen (a-d), and in metaphase, the chromosomes were aggregated and could be seen in the equator of the cell (e). In anaphase, the daughter chromosomes separated, and in telophase the cytoplasm had an elongated shape, the cleavage furrow started to appear, and the nuclear membranes emerged and the cytoplasm began to constrict in the middle (f). However, the constriction stopped and reversed in the middle of cytokinesis, and the cytoplasm was not divided. As a result, the cell, which included two nuclei, was found in one cytoplasm (g-i).

**Figure 6:**
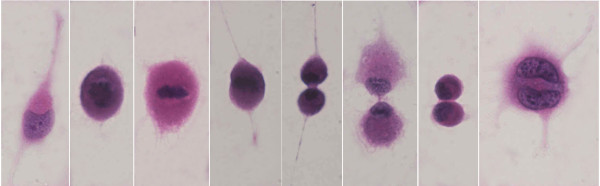
**HE staining models corresponding to the time-lapse images of Figure 5**.

As for the dynamics of multinucleation, the mononuclear cell moved about extensively, and extended some protrusions. The mononuclear cells were not so much spindle shaped as amoebiform and were round in shape with some protrusions. At the onset of M phase, the nuclear body and the nuclear membrane were disaggregated and could not be seen (prophase), and then the chromosomes were aggregated and could be seen in the equator of the cell (metaphase). The protrusions receded and the cytoplasm changed spherically, and almost floated. The daughter chromosomes separated (anaphase) and, simultaneously, the cytoplasm developed an elongated shape, the cleavage furrow started to appear, the nuclear membranes emerged, and the cytoplasm started to constrict in the middle (telophase). However, the constriction stopped and reversed in the middle of cytokinesis, and the cytoplasm did not divide. As a result, the cell, which included two nuclei, contained one area of cytoplasm (Figure 5; Additional file 2). Similar states were found in the hematoxylin and eosin staining, although each image is presented as a distinct cell (Figure 6). Multinucleation was also observed in a different process between telophase and cytokinesis, such as before the appearance of the cleavage furrow or at the end of the constriction.

Almost all of the multinucleated cells developed as described above, and only one cell seemed to be fused to another cell. The cytoplasm of the multinucleated cell was more amoebiform and irregular in shape than that of the mononuclear cell.

## Discussion

There are two mechanisms which are considered to result in multinucleation; the cell-cell fusion process and acytokinetic cell division [12]. Various multinucleated cells have been investigated. The multinucleated cells in normal tissue are considered to be formed by cell-cell fusion, for the most part [1,13].

As for the neoplastic multinucleated cells, Reed-Sternberg cells are well known and extensively reported in the past literature. In a recent study, it is suggested that multinucleation involves acytokinetic cell division rather than cell-cell fusion [14-16]. However, there is no direct evidence for the processes of acytokinesis and multinucleation in other neoplasms.

Ki-67 is a 395-kDa nuclear antigen, and the expression is confined to late G1, S, M, and G2 growth phases [17]. A simple and rapid determination of the growth fraction of a given human cell subset has become possible with the help of Ki-67 [18]. The expression of Ki-67 indicates DNA synthesis and nuclear division [14]. In our study, a Ki-67 immunohistochemical analysis revealed a high positive rate and a mitotic ability of the multinucleated cells, thus suggesting the occurrence of acytokinetic multinucleation. But these findings can not rule out the presence of a cell-cell fusion mechanism.

The time-lapse live-cell imaging enabled us to search the dynamic state or mitotic form of the actual cells using a non-invasive approach. There are no reports on multinucleation of myxofibrosarcoma being observed by the use of dynamic images. In this *in vitro *study, we successfully visualized imperfect cell division which led to multinucleation. Furthermore, Ki-67 was positive for multinucleated cells of the parental tumors and xenografts. These results may reflect the multinucleation of the *in vivo *myxofibrosarxoma tissue. Multinucleation almost arose during the process beginning with the appearance of the cleavage furrow to the end of the constriction. A contractile ring, which is mainly composed of actin and myosin II, plays an important role in this process. The diameter of the contractile ring decreases, so that the cell is pinched into two parts by a deepening cleavage furrow, from telophase to cytokinesis [19].

In addition, the cytoplasm of the multinucleated cell seemed to be irregular and feeble. These findings suggest an aberrance of the cytoskeleton structure.

These results indicate that vulnerability of the cytoskeleton components causes multinucleation. In malignancy, some gene abnormalities are frequently observed, and there is a possibility that an abnormality of a gene relating to the cytoskeleton components causes the formation of multinuclear cells.

In this study, we investigated only the myxofibrosarcoma cells. Therefore, the mechanism of multinucleation in other types of malignant cells remains unclear. In future studies, other malignant cell types must be examined by time-lapse microscopy.

## Competing interests

The authors declare that they have no competing interests.

## Authors' contributions

All the authors contributed as mentioned. TA and AO conceived of the study and wrote the manuscript. HK, TH and NE participated in the design of the study and helped write the paper. HU gave pathological suggestion to this work.

## Supplementary Material

Additional file 1 **Dynamics of normal cell division by time-lapse video microscopy.** Click here for file

Additional file 2 **Dynamics of multinucleation by time-lapse video microscopy.** Click here for file
